# Level of optimal antenatal care utilization and its associated factors among pregnant women in Arba Minch town, southern Ethiopia: new WHO-recommended ANC 8^+^ model

**DOI:** 10.3389/fgwh.2024.1259637

**Published:** 2024-07-16

**Authors:** Dagne Deresa Dinagde, Gizu Tola Feyisa, Hana Tadesse Afework, Menen Tilahun Chewaka, Habtamu Wana Wada

**Affiliations:** ^1^ Department of Midwifery, College of Health Sciences, Mattu University, Mattu, Ethiopia; ^2^ Department of Midwifery, College of Medicine and Health Sciences, Mizan Tepi University, Mizan Tepi, Ethiopia; ^3^ Department of Midwifery, Health Sciences College, Arba Minch, Ethiopia

**Keywords:** optimal antenatal care, ANC 8^+^ model, Arba Minch town, southern Ethiopia, optimal antenatal

## Abstract

**Background:**

To fully realize the life-saving and health-promoting benefits of antenatal care (ANC), the latest World Health Organization (WHO) recommendations call for pregnant women to have at least eight contacts with skilled healthcare providers. This increased number of recommended ANC visits represents a shift toward a more comprehensive, individualized approach to prenatal care. The focus is on health promotion, disease prevention, and the early detection and management of complications during pregnancy. However, in sub-Saharan African countries, including Ethiopia, the coverage rate for this level of recommended antenatal care is only 58%. Given this relatively low utilization, identifying the key risk factors that prevent adequate antenatal care would have significant implications for increasing overall ANC uptake in these regions.

**Objective:**

The aim of the present study was to assess the level of optimal antenatal care utilization and its associated factors among pregnant women in Arba Minch town, southern Ethiopia in 2023 using the new WHO-recommended ANC 8^+^ model.

**Methods:**

An institution-based cross-sectional study was conducted among 416 mothers who were enrolled between 1 December 2022 and 30 January 2023. The total sample size was allocated proportionately to the number of women who delivered at each public health facility. Thus, systematic sampling was applied. Kobo Toolbox was used for data collection and cleaning, which was then analyzed using SPSS Version 26. Statistical significance was determined at a *p*-value <0.05.

**Results:**

In this study, the level of optimal antenatal care was 41% [95% confidence interval (CI): 37–45.3]. The associated factors with optimal antenatal care were the presence of pregnancy danger signs [adjusted odds ratios (AOR) = 4.1, 95% CI: 1.87–8.82], having bad obstetric history (AOR = 3.90, 95% CI: 1.94–7.83), antenatal contact at hospital (AOR = 5.11, 95% CI: 2.28–11.21), having good knowledge about antenatal care (AOR = 2.26, 95% CI: 1.15–4.44), women's high decision-making power (AOR = 3.9, 95% CI: 1.2–7.63), and male partner involvement (AOR = 2.0, 95% CI: 1.04–3.78) were positively associated with optimal antenatal care utilization.

**Conclusion:**

The level of optimal antenatal follow-up is still low. Therefore, it is crucial to provide more information during the antenatal contacts to lower the rate of women discontinued from antenatal care.

## Introduction

Antenatal care (ANC) is a maternal healthcare service provided by coordinated healthcare professionals to pregnant women to support and maintain their optimal health during pregnancy, delivery, and puerperium, as well as to have and raise a healthy baby. It also provides an opportunity for nutrition, birth preparation, delivery care, and postpartum contraceptive education (1).

The World Health Organization (WHO) has recommended the four-visit ANC model since 2002, which recommends at least four visits, but an updated version with eight ANC contacts was released in 2016 that calls for ANC contacts before 12 weeks of gestation. The word “visit” in the previous model has now changed to “contact” to indicate an active interaction between a pregnant woman and a healthcare provider (1) and the new model is being implemented since Ethiopia adopted the new recommendation and incorporated it in the management protocol for hospitals in 2021 (2).

Women who did attend each of the suggested contacts of ANC are considered to have optimal (adequate) antenatal care (3). Particularly in low-resource settings, women who receive skilled care during pregnancy often “drop out” during a critical period (pregnancy) of care and end up delivering at home or in the community without a certified, properly trained health professional, putting themselves and their newborns at risk (4). Many maternal and prenatal deaths occur in women who have received inadequate and no utilization of ANC (5).

In Africa, the coverage of antennal care was only 58% (6), with west and central Africa having the lowest ANC coverage (53%) (7). According to the Ethiopia Demographic and Health Survey (EDHS) 2019, the percentage of women who received at least one ANC visit by a skilled professional was 74% and for those who received four or more was 43% (8).

Before the introduction of antenatal care by the WHO in the 2000s, maternal mortality rates (MMRs) were at historically high levels globally. In the 1990s, there were 400 maternal deaths worldwide per 100,000 live births, which today has decreased to 223 deaths per 100,000 live births (9, 10). It has decreased maternal mortality by 34% over two decades However, globally, a woman is still dying every 2 min from almost all preventable causes, which is 800 mothers every day (5).

To combat this, WHO and other stakeholders are working to reduce maternal and child mortality through various intervention programs and strategies, such as through preventable deaths, ensuring good health and wellbeing (11) and the Sustainable Development Goals (SDG) plan to decrease maternal mortality to below 70 maternal deaths per 100,000 live births by 2030 (12).

There is a program that uses Health Extension Workers (HEWs) to mobilize the community to increase attendance at ANC visits. The goal is to provide health education on the advantages of regularly attending ANC appointments, as well as the burdens and risks associated with not visiting for ANC. The program aims to raise awareness and encourage pregnant women to prioritize and attend their recommended ANC checkups.

A different cross-sectional study conducted worldwide revealed that sociocultural and economic barriers, poor access to health services, long distance from healthcare facility services, lack of knowledge, lack of professional advice, poor wealth index, and not developing a danger sign were factors negatively associated with optimal ANC (13–15).

However, in this study, variables such as type of institution, perceived quality of care, level of respectful and non-abuse care and partner involvement were additionally included in this study. Even though some studies have been conducted on the factors contributing to optimal antenatal care utilization in Ethiopia (16, 17), after the introduction of the recommended new ANC model, there is some information and certainly undiscovered factors that were revealed within this study. Therefore, the objective of the present study was to assess the level of optimal antenatal care and the factors related to adequate contacts of antenatal care during pregnancy in Arba Minch town.

## Materials and methods

### Study design and setting

An institutional cross-sectional study was conducted among postpartum mothers who gave birth at health facilities in Arba Minch town between 1 December 2022 and 30 January 2023. The town is located 505 km southwest of Addis Ababa, the capital city of Ethiopia, and 275 km away from Hawassa, the commercial and administrative center of the southern region. According to the 2022 population projection, Arba Minch town has a total population of 201,049 (101,130 male, 100,019 female) (18). The town has two public hospitals, one private hospital, and two health centers. All the health facilities provide perinatal care, with 15 nurses and 30 midwives providing antenatal care in those health facilities. The public health institutions in the town are expected to serve more than half a million people in the town and nearby districts (19).

### Study participants

The study participants were mothers initially booked for antenatal care visits and who gave birth in public health facilities in Arba Minch town and were available during the data collection period. Those willing to give information were included while those mothers critically ill on the day of data collection and referred from other facilities outside Arba Minch were excluded from the study.

### Sample size determination

The sample size was calculated using a single population proportion formula: $n=(\mathrm{za}{)}^{2}\frac{\;p(1-p)}{d^{2}}$, considering the following assumptions: the magnitude optimal antenatal care utilization analysis from Ethiopian EDHS (43%) (20), with a 95% confidence interval (CI), and 5% margin of error.

$$n=(\mathrm{za}{)}^{2}\frac{\;p(1-p)}{d^{2}}$$

$$n=(1.96{)}^{2}\frac{0.43(1-0.57)}{0.05^{2}}=378$$

With a 10% non-response rate included, a final sample size of 416 was obtained.

### Sampling technique and procedure

**Figure 1:**
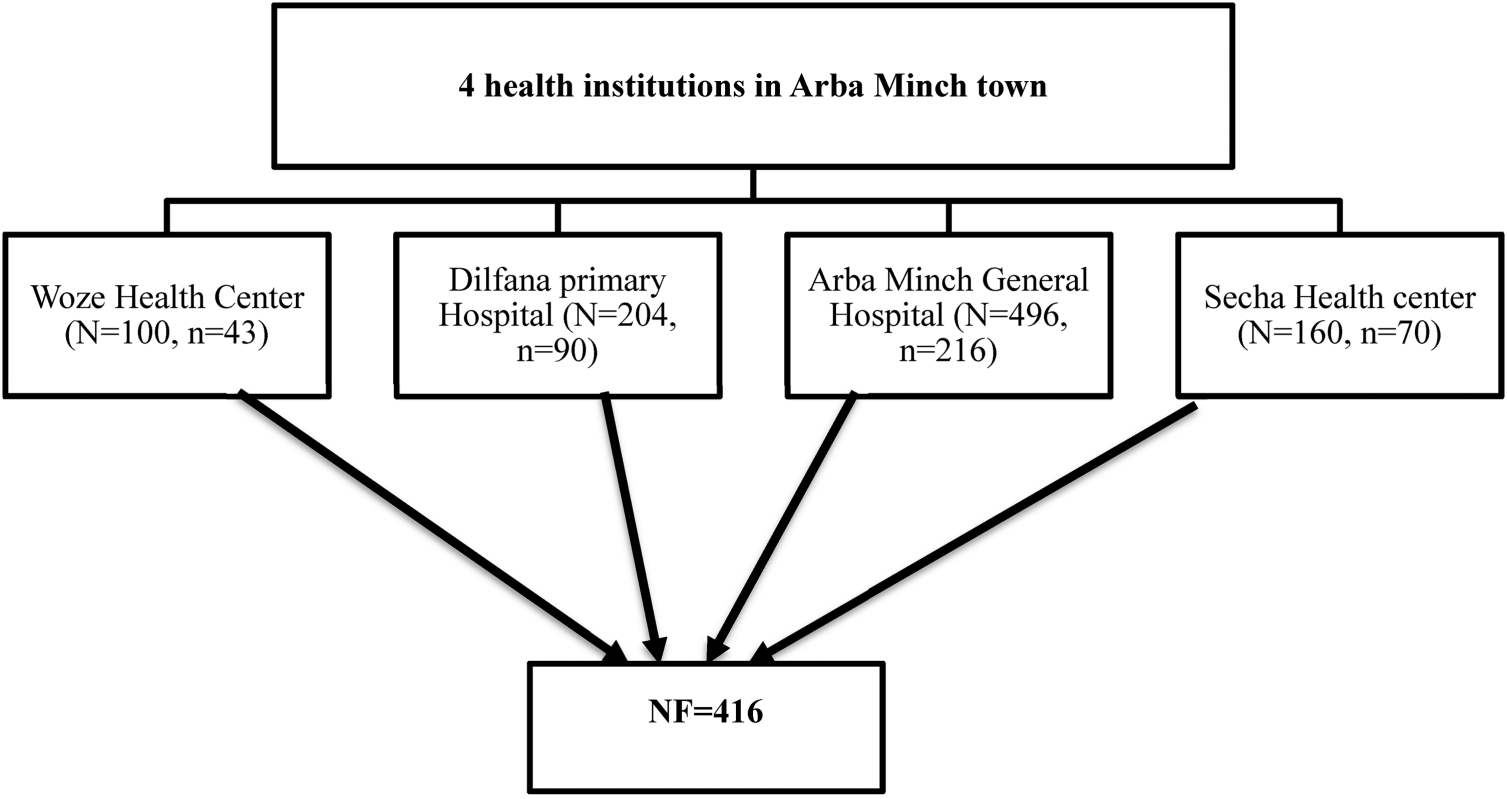
A schematic diagram shows the proportional allocation of the sample size of each health institution in Arba Minch town.

Two public hospitals and two public health clinics serve the population by offering both curative and preventive services in Arba Minch town. This study considers all public health centers and hospitals in the town. The mothers were then enrolled if they gave birth at these healthcare institutions. The systematic sampling technique was used to determine the sample size for four healthcare facilities. As a result, the total numbers of participant from all four facilities was determined using the delivery registration book from the previous year for the same months (i.e., December to January). According to the 2021 report of each health facility's annual skilled birth attendant report, at similar times in the past 2 months, 496 women delivered in Arba Minch General Hospital, 204 mothers gave birth in Dilfana Primary Hospital, 160 women delivered in Secha Health Center, and 100 women gave birth in Woze Health Center. These women were used as the sampling frame. The respondents were then found using a systematic random sampling method. As a result, the first respondent was chosen by lottery among the first k intervals, and the other research participants were chosen by every ‘k’th (i.e., every second) value for all institutions until the required total sample size was reached (i.e., 416) (Figure 1).

### Data collection tool and data quality management

The questionnaire used was an interviewer-administered semi-structured questionnaire adapted from relevant literature (13, 21–25) with modifications and employed for data collection. The tool consists of four sections: sociodemographic, institutional, personal factors, and reproductive variables. The questionnaire was designed in the English and Amharic languages. Women who gave birth in public health facilities in Arba Minch town were enrolled by applying systematic sampling depending on case flow of the same 2 months of the previous year, using it as a sampling frame. Pre-testing of the questionnaire was carried out 2 months before the commencement of the data collection among 20 mothers who gave birth in Shalle Health Center and all the necessary corrections were made based on the pretest result to avoid any confusion and for better completion of the questions. Then, four nurses and three midwives were recruited for data collection based on their past experience and fluency in the local language, and they were supervised by two master holder midwives. Checking for double data entry, consistency, missing values, and outliers was done by the supervisors and principal investigator, and comments and measures were undertaken throughout the data collection period.

### Data analysis and entry

The data were coded, collected, cleaned, and entered using Kobo Toolbox and exported to Statistical Package for Social Science (SPSS) Version 26 for analysis. Inconsistencies and missing values were checked by running frequencies and other data explorations. Descriptive statistics, such as frequency distributions and mean and standard deviation, were computed. A bivariate analysis was carried out primarily to check which independent variables had an association with that of the dependent variable. Independent variables with marginal associations (*p* < 0.25) in the bivariate analysis, which are biologically plausible and showed significant association in the previous studies, were entered into a multivariate logistic regression analysis to detect the association with antenatal dropout. The multicollinearity was checked among independent variables and the Hosmer–Lemeshow test was used to check the appropriateness of the model for analysis. Finally, adjusted odds ratios (AOR) and a 95% CI were estimated to assess the strength of associations and statistical significance was declared at a *p*-value <0.05. The results were presented using tables, figures, and texts.

## Study variables

### Dependent variable

The dependent variable was optimal antenatal care (Yes/No).

### Independent variables

Independent variables were as follows: demographic factors, such as maternal age, level of education, marital status, parity, employment status, woman's occupation, husband's educational status, husband's occupation and residence; health facility related, such as insurance status, distance to health facility, cost of transportation, and dissatisfaction with the information provided during ANC visit, type of institution, waiting time, and counseling on danger signs, level of respectful and non-abuse care; reproductive (obstetric) factors, such as desire for pregnancy, previous mode of delivery, parity, danger signs, and bad obstetric history (BOH); and personal factors, such as attitude, client's satisfaction, client knowledge, and partner support.

### Operational definition and measurements

Optimal antenatal care: women were regarded to have optimal antenatal care if they attended each suggested visit according to the new WHO recommendation of 2016 or on another hand it included those neither have delay registration of ANC nor discontinue from the services (3).

Knowledge of ANC: the overall level of antenatal care knowledge was evaluated by scoring responses that measured participants’ knowledge based on the descriptions below: a score of 1 was assigned if the participant had knowledge and a score of 0 if they did not. A total score and a mean score were computed, with a score less than the mean indicating poor knowledge and a score equal to or higher than the mean indicating good knowledge (26).

Attitude toward ANC was measured using a 4-point Likert scale (1 = strongly agree, 2 = agree, 3 = disagree, and 4 = strongly disagree). Positive attitude was assigned for those scored above the mean and negative attitude was assigned if they scored below the mean (27).

Women's decision-making power: this was one of the key indicators that measure the level of women's involvement in household decision-making regarding consumption, expenditure, and reproductive choices. Decision-making power was labeled as high if the mother was involved in making decisions independently or with others and labeled as poor if she was never involved in decision-making (28).

Distance from the facility: long distance was defined as taking more than 60 min to reach the health facility and short distance was defined as taking less than 30 min (22).

Types of institution: hospital, health center, or health post to which they preferred to go.

Partner support: partner support was assessed using a modified eight-item Spousal Support Scale (SSS) that was customized based on the local context. SSS scores were in the range of 8–48, and reverse scoring was used (5 = strongly agree to 1 = strongly disagree). A score higher than the mean value indicated that the respondent had a positive perception of partner support (29, 30).

Health insurance status: this was categorized as mothers with or without an insurance card that allowed them free health services (13).

Waiting time: a prolonged waiting time was defined as waiting for services for >30 min and not prolonged if receiving services within 30 min (31).

## Results

### Sociodemographic characteristics of study participants

All participants (*n* = 416) were actually interviewed and provided accurate information, yielding a response rate of 100%. The mean age of the women was 29.7 ± 6 years (range 18–42 years). The majority of the participants (*n* = 276, 52.9%) were Orthodox Christians. The majority of the women (*n* = 204, 49%) were housewives and 164 (39.4%) could read and write. Of the study participants, 364 (83.2%) had a monthly income of more 5,000 Ethiopian birr (Table 1).

**Table 1:** Sociodemographic characteristics of study participants in Arba Minch town (*n* = 416), 2023.

Variables	Response	Frequency	Percent (%)
Age(in years)	<20	7	1.7
** **	20–24	90	21.6
	25–29	103	24.8
	30–34	88	21.2
	35 and above	128	30.8
Residence	Rural	130	31.3
	Urban	286	68.8
Maternal education	Unable to read and write	99	23.8
	Able to read and write	164	39.4
	Primary	91	21.9
	Secondary school and above	62	14.9
Maternal occupation	civil servant	81	19.5
	Farming	23	5.5
	house wife	204	49.0
	Traders	108	26.0
Husband education	Unable to read and write	87	20.9
	Able to read and write	175	42.1
	Primary	64	15.4
	Secondary school and above	90	21.6
Husband occupation	Farming	151	36.3
	Traders	96	23.1
	civil servant	168	40.4
Income (ETB)	<2,500	13	3.1
	2,501–5,000	57	13.7
	>5,000	346	83.2

### Facility-related factors

Of the participants, 205 (73.3%) stated that they waited longer than 30 min to get the ANC service. Of the respondents, 37 (8.9%) said that they traveled more than 1 h to get the ANC service, and 121 (29.1%) traveled to the health center on foot and 29 (9%) were transported by mule or horse. The majority of women (81.5%) who traveled by car or motor paid more than 20 Ethiopian birr to arrive at a medical facility and receive ANC care. The majority (82.2) had a previous history of ANC visits. Only 46 (11.1%) respondents said that they were not counseled about ANC services during a previous pregnancy by health professionals and only 14 (3%) mothers reported that they had experienced a good level of respect and non-abusive care (Table 2).

**Table 2:** Facility-related factors of ANC among study participants in Arba Minch town, southern Ethiopia, 2023 (*n* = 416).

Variables	Response	Frequency	Percentage
Distance in time	≤30 min	42	10.1
	30–60 min	337	81.0
	>1 h	37	8.9
Mode of transportation	Horse/on foot	150	36.1
	By car	266	63.9
Cost of transport (ETB)	≤20	77	18.5
	>20	339	81.5
Advice about ANC	Yes	370	88.9
	No	46	11.1
Waiting time (min)	≤30	111	26.7
	>30	305	73.3
History of ANC	Yes	342	82.2
	No	74	17.8
Level of respect	Good level of respect	14	3.4
	Poor level respect	402	96.6

### Reproductive and personal factors

Most mothers (*n* = 358, 84%) were multiparous and had more than two children, and 306 (73.6%) mothers said the pregnancy was wanted. Most mothers (270, 81%) gave birth through spontaneous vaginal delivery (SVD) previously, and 129 (31%) women had a history of bad obstetrics, such as stillbirth, congenital anomalies, abortion, and neonatal death. Of the mothers, 49 (21.9%) had a history of pregnancy danger signs and some complications. The majority (*n *=* *342, 82.2%) had a previous history of antenatal care. Overall, 238 (57.2%) study participants had high decision-making autonomy for the utilization of maternal and neonate healthcare services (Table 3).

**Table 3:** Reproductive and personal factors of ANC among study participants in Arba Minch town, southern Ethiopia, 2023 (*n* = 416).

Variables	Options	Frequency (*n*)	Percentage
Insurance card	Yes	265	63.7
** **	No	151	36.3
Parity	Primipara	67	16.1
** **	Multipara	349	83.9
Wanted pregnancy	Yes	306	73.6
** **	No	110	26.4
Had history of ANC	Yes	342	82.2
	No	74	17.8
Mode of delivery	SVD	270	81
** **	C/S	63	19
Media exposure	Yes	321	77.2
** **	No	95	22.8
Bad obstetric history	Yes	138	33.2
** **	No	278	76.8
Pregnancy danger signs	Yes	91	21.9
** **	No	325	78.1
Knowledge of ANC	Good knowledge	212	51
	Poor knowledge	204	49
Patients’ attitude	Positive attitude	241	57.9
	Negative attitude	175	42.1
Partner support	Good	231	55.5
	Poor	185	44.5
Women autonomy	Yes	178	42.8
	No	238	57.2

### Level of optimal ANC

**Figure 2:**
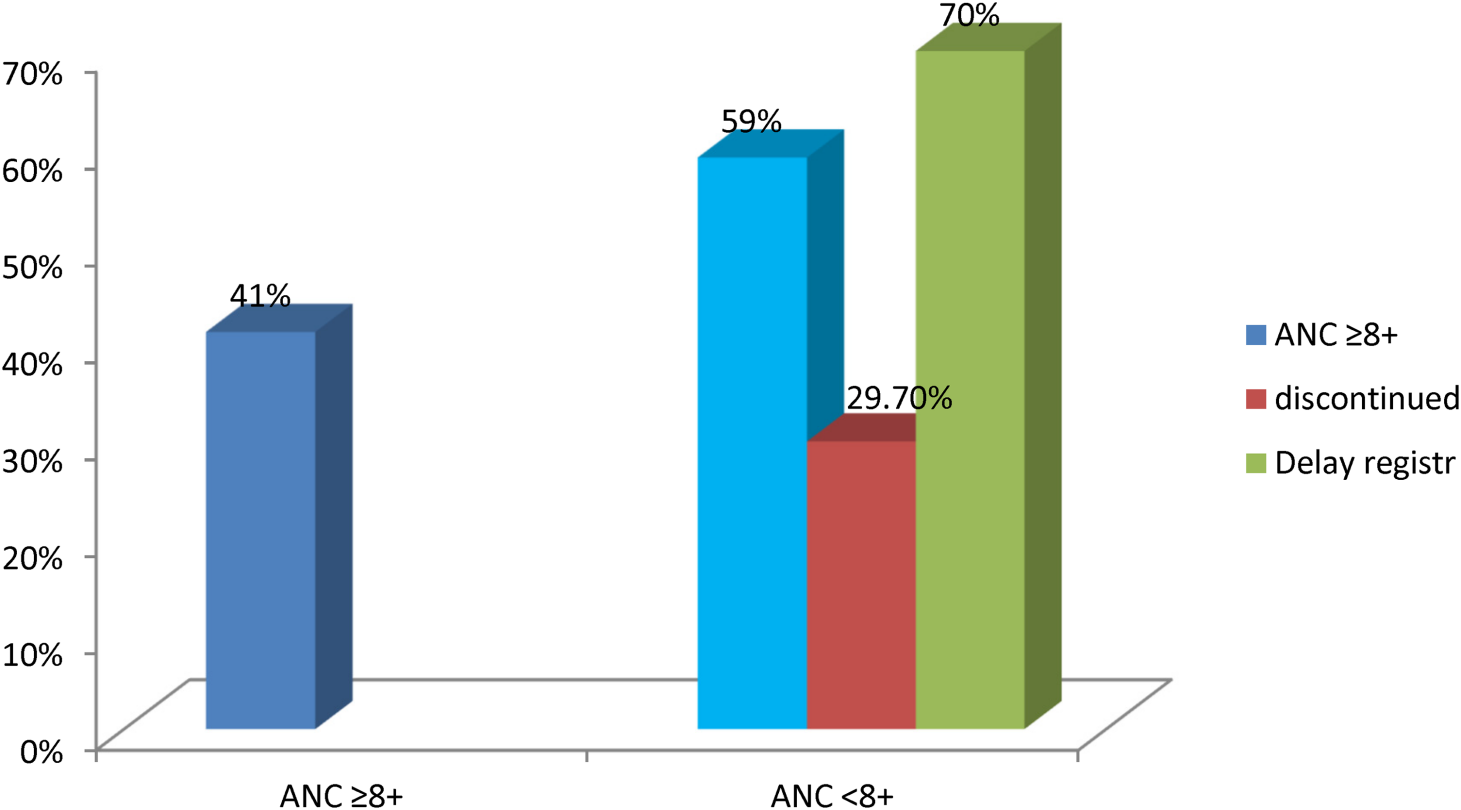
The level of optimal antenatal care among study participants at Arba Minch town, southern Ethiopia, 2023 (*n* = 416).

From a total of 416 women booking an ANC follow-up, the magnitude of optimal ANC was 170 (41%, 95% CI: 37–45.3): 246 (59%) were found to either discontinue (29.7%) or delay registration (70.3%) as assessed according to the modified WHO recommendation (Figure 2).

### Factors associated with optimal ANC

Assumptions for logistic regression, such as large enough sample (minimum of 10 observation for each independent variables), residuals (>3), normal distribution [Shapiro Wilk's test (*p* > 0.05) and visual inspection of histogram, normal Q-Q plots, and box plots] and no multicollinearity [with Spearman correlation* *<* *0.7 or collinearity diagnosis (tolerance* *>* *0.1 and variance inflation factor (VIF) < 10)] before categorization of variables were checked. Finally, the Hosmer–Lemeshow test was checked for model fitness (sig = 0.418).

In the bivariate binary logistic regression, those variables with *p* < 0.25 were candidates for multiple logistic regression and statistical significance was declared at a *p*-value < 0.05 as below. Thus, age, maternal education, maternal occupation, marital status, parity, religion, type of institution, mode of transport, husband’s education, husband’s occupation, distance, counsel on ANC, waiting time, having community insurance card, income, unwanted pregnancy, exposure to media, BOH, pregnancy danger signs, patients’ attitude toward ANC, partner support, level of respect, women's autonomy, and knowledge about ANC were candidates for multivariate analysis.

This study's multivariable logistic regression analysis revealed that types of health facilities, parity, bad obstetric history, pregnancy danger signs, patients’ knowledge, partner involvement, and women's autonomy all had a statistically significant association with the outcome variable, optimal ANC (Table 4).

**Table 4:** Multivariate logistic regression analysis results for variables associated with optimal ANC among pregnant mothers who gave birth at Arba Minch health facilities (*N* = 416).

Variables		Optimal ANC		COR (95% CI)	AOR (95% CI)	*p*-value
		Yes (%)	No (%)			
Health facility	Center	20 (18.2)	90 (81.8)	1	1	
	Hospital	150 (49)	156 (51)	4.33 (2.54–7.38)	5.1 (2.28–11.21)	0.000*
BOH	Yes	82 (59.4)	56 (40.6)	3.16 (2.07–4.83)	3.9 (1.94–7.83)	0.000*
	No	88 (31.7)	190 (68.3)	1	1	
Pregnancy danger signs	Yes	64 (71.1)	27 (29.7)	4.89 (2.92–8.14)	4.1 (1.87–8.82)	0.000*
	No	106 (32.6)	219 (67.4)	1	1	—
Partner involvement	Yes	98 (42.4)	133 (57.6)	5.43 (3.62–8.19)	2.0 (1.04–3.78)	0.037*
	No	148 (80)	37 (20)	1	1	
Women's autonomy	Autonomous	31 (17.4)	147 (82.6)	1	1	—
	Not autonomous	139 (58.4)	99 (41.6)	6.65 (4.65–8.21)	3.9 (1.2–7.63)	0.000*

1, Reference group; COR, crude odd ratio.

* *p* < 0.05 (statistically significant).

The types of health facilities had a positive association with optimal ANC, with mothers attending their ANC services at hospital being five times more likely to receive adequate ANC than those attending services at health centers (AOR = 5.1, 95% CI: 2.28–11.21).

Furthermore, mothers who did have a BOH were four times more likely to receive optimal ANC contact compared to those who did not have a BOH, such as still birth, congenital anomaly, neonatal death, and recurrent abortion (AOR = 3.90, 95% CI: 1.94–7.83). Knowledgeable mothers were found to consume ANC services. Those with good knowledge were twice as likely to receive an ANC follow-up compared to their counterparts (AOR = 2.26, 95% CI: 1.15–4.44). Mothers who did experience any danger signs during this pregnancy were four times more likely to obtain full ANC than those who did not (AOR = 4.1, 95% CI: 1.87–8.82).

Mothers with high decision-making power were 3.9 times more likely to have optimal ANC contacts according to schedules (AOR = 3.9, 95% CI: 1.2–7.63).

Those mothers whose husbands were involved in ANC were twice as likely to have optimal antenatal care compared to those without support from their partners (AOR = 2.0, 95% CI: 1.04–3.78).

## Discussion

The present study revealed that the level of optimal antenatal care was 41%. This finding is in line with a study of the general population (43%) (32). However, this finding is higher than in a study conducted in Debra Tabor, northwest Ethiopia (35.3%) (33). Tanzania revealed similar results, where only 10% of mothers received the recommended antenatal care follow-up (34), along with a study conducted in Kilifi town, Kenya (32%) (35). However, this finding is lower than in a study conducted in Nigeria in 2016 (62%) (36). This may be acceptable given that all the research mentioned above provided data using focused ANC, which was easy to return and had a low dropout rate.

Women's knowledge of ANC is crucial in the utilization of ANC services during pregnancy. This finding revealed that those mothers with a good knowledge of ANC were about twice as likely to receive more ANC visits compared to those with a poor knowledge of ANC. This is in line with studies conducted in Pakistan (37), Ghana (26), Somalia (38), and Gonder town, Ethiopia (39). It is known that women who are knowledgeable about ANC services are more likely to comprehend and appreciate the services offered during ANC.

The odds of optimal antenatal care among those women with BOH were four times higher compared to those without bad obstetric history. This finding is supported by a study conducted in Rwanda (40). Women may be afraid of a recurrence, which could be the cause of bad outcome.

The use of adequate antenatal care services was substantially correlated with the development of danger signs during pregnancy. Women who did experience a danger sign during their pregnancy were four times more likely to continue attending prenatal care appointments than those who did not. This result is in line with studies conducted at Bahir Dar Zuria (21) and Shashemane, south Ethiopia (41), which indicates that mothers were more likely to use the services when they were aware of pregnancy risk factors. Because they were worried about the repercussions, they might not have ever skipped an appointment.

The completion of the ANC follow-up was statistically and favorably related to having authority over medical decision-making. The odds of those mothers with optimal ANC follow-up were four times higher among those mothers with high decision-making power compared to their counterparts. This finding was consistent with that from Pakistan (42) and northwest Ethiopia (23). This may be because women with control over healthcare decisions may have greater mobility, fewer financial concerns, and the ability to travel independently to receive care. In addition, there may be a relationship between autonomy and other factors, including women's education and urban residence, both of which are related to an increased likelihood of using maternal healthcare.

This study also found that greater involvement of a male partner was seen among women receiving complete ANC follow-ups per the schedule. Women with support from their partners were twice as likely to receive all recommended antenatal follow-ups when compared to their counterparts. This finding was supported by studies conducted in Gulu district, Uganda (43), and Addis Ababa, Ethiopia (30, 44). A potential reason for the link could be that when male partners become more involved, their knowledge grows and they adopt a more favorable attitude toward maternal health services.

Types of health institution at which they were receiving ANC services have to be positively correlated with adequate ANC. Those mothers receiving their antenatal care at hospitals were five times more likely to obtain recommended ANC visits compared to those with an ANC follow-up at health centers. This may be justified as more sophisticated services and counseling are given at the hospital level with better health professionals.

### Strengths and limitations of the study

The sample size employed in this study supports the generalizability of the results to all women in the study area who are of reproductive age. Information in the survey is based on self-reports, so there may be social desirability bias and recall bias. Thus, medical record cards are in parallel checked to minimize recall bias. In addition, the entire literature review for the study was conducted according to focused antenatal care, which reduced the accuracy of the comparability.

## Conclusion

This study showed that the study area had a low level of optimal antenatal care. Bad obstetric history, danger signs of pregnancy, ANC follow-up at hospital, as well as women's autonomy, male partner involvement, and good knowledge are factors associated with optimal ANC. Therefore, it is important to provide more information during the antenatal contacts to increase the rate of women receiving all eight recommended visits (contacts).

## Data Availability

The original contributions presented in the study are included in the article/**Supplementary Material**, further inquiries can be directed to the corresponding author.
