# Supplementary material for: Level of optimal antenatal care utilization and its associated factors among pregnant women in Arba Minch town, southern Ethiopia: new WHO-recommended ANC 8+ model
Source: Front Glob Womens Health. 2024 Jul 16;5:1259637. doi: 10.3389/fgwh.2024.1259637 (PMC11286477; doi:10.3389/fgwh.2024.1259637)
Supplement: Supplementary file 1 [file Table1.docx]

## Annex V:-English Version Questionnaires

Health facility of ANC ------------------------

| NO | **.Socio-demographic factors** |  | | | | | | | | | | | | | | | |
| --- | --- | --- | --- | --- | --- | --- | --- | --- | --- | --- | --- | --- | --- | --- | --- | --- | --- |
| 101 | How old are you? | In years ……………….. | | | | | | | | | | | | | | | |
| 102 | Residence | 1. Rural 2. Urban | | | | | | | | | | | | | | | |
| 103 | Monthly income | ---------------ETB | | | | | | | | | | | | | | | |
| 104 | Your religion? | 1. Orthodox 2. Muslim 3. Protestant   Others specify……….. | | | | | | | | | | | | | | | |
| 106 | Your educational level? | 1. Unable to read and write 2. Able to read and write 3. Primary 4. Secondary school & above | | | | | | | | | | | | | | | |
| 107 | Your occupation? | 1. House wife 2. Farming 3. Traders 4. Civil servant   Other specify …………. | | | | | | | | | | | | | | | |
| 108 | Your marital status? | 1. Single 2. Married but Separate 3. Married and live to together 4. Divorced 5. Windowed | | | | | | | | | | | | | | | |
| 109 | If married, Your husband’s educational status? | 1. Unable to read and write 2. Able to read and write 3. Primary 4. Secondary school & above | | | | | | | | | | | | | | | |
| 110 | If married, Your husband’s occupation? | 1. Farming 2. Traders 3. Civil servant   Other specify ………….. | | | | | | | | | | | | | | | |
|  | **Health Facility related factors** |  | | | | | | | | | | | | | | | |
| 201 | How many hours you travel to reach for the health services? | By minutes ---------------- | | | | | | | | | | | | | | | |
| 202 | What’s your means of transportation to reach ANC services? | 1. On foot 2. On the back of the horse/mule 3. By car, cart | | | | | | | | | | | | | | | |
| 203 | How much you paid to reach health services? | --------------ETB | | | | | | | | | | | | | | | |
| 204 | How much you wait to get the services? | --------------- minute | | | | | | | | | | | | | | | |
| 205 | Have you receive ANC in previous pregnancy? (multipara only) | 1.Yes  2. No | | | | | | | | | | | | | | | |
| 206 | Have you ever counseled about ANC service by health professional? | 1. Yes 2. No | | | | | | | | | | | | | | | |
|  | **Personal & reproductive related factors** |  | | | | | | | | | | | | | | | |
| 401 | How many times have you been pregnant? | 1. Once 2. 2 up to 5 3. More than 5 | | | | | | | | | | | | | | | |
| 402 | Would the last pregnancy is wanted | 1. Yes 2. no | | | | | | | | | | | | | | | |
| 403 | Numbers of children? | ……. | | | | | | | | | | | | | | | |
| 404 | Mode of delivery for previous pregnancy (multipara only) | 1. SVD 2. C/S | | | | | | | | | | | | | | | |
| 405 | How many times did you receive antenatal care during this Pregnancy? | ------------------------ | | | | | | | | | | | | | | | |
| 207 | Have you carded for community health insurance? | 1. Yes 2. no | | | | | | | | | | | | | | | |
| 407 | **From pt. card ( recommended contacts)** |  | | | | | | | | | | | | | | | |
| - | 1st visit (up to 12wks) | 1.Yes 2.No | | | | | | | | | | | | | | | |
| - | 2nd visit (13-20 wks.) | 1.Yes 2.No | | | | | | | | | | | | | | | |
| - | 3rd visit (21wks -26 wks.) | 1.Yes 2.No | | | | | | | | | | | | | | | |
| - | 4th visit (27 wks-30 wks.) | 1.Yes 2.No | | | | | | | | | | | | | | | |
|  | 5th visit (31wks-34 wks.) | 1.Yes 2.No | | | | | | | | | | | | | | | |
|  | 6th visit (35 wks-36 wks.) | 1.Yes 2.No | | | | | | | | | | | | | | | |
|  | 7th visit (37wks-38 wks.) | 1.Yes 2.No | | | | | | | | | | | | | | | |
|  | 8th visit (39 wks-40 wks.) | 1.Yes 2.No | | | | | | | | | | | | | | | |
| 408 | If there is any lost recommended contact from 407, why? | 1. Delay registration  2. Discontinued | | | | | | | | | | | | | | | |
| 409 | If they discontinue, Why you are discontinue | 1. They don’t tell me when to come back 2. I feel health 3. Due to distances 4. Being busy 5. The services was not attracting   Other specify …………… | | | | | | | | | | | | | | | |
| 408 | Which type of media you more used? | 1. Radio  2. TV  3. No  4. other------------ | | | | | | | | | | | | | | | |
| 410 | Have you experienced any of the following during pregnancy ***(obstetric complications)*** | 1. Vaginal bleeding 2. Leakage of liquor 3. Severe headache not responsive to analgesics 4. Blurred of vision 5. ,high grade Fever 6. Lower Abdominal pain 7. Epigastric burning 8. RUQT 9. Foully smelling vaginal discharged 10. Convulsion 11. Not at all 12. Others---- | | | | | | | | | | | | | | | |
| 413 | Have you History of(BOH) PTL, Still birth, early neonatal loss, or congenital anomaly | 1. Yes 2. No | | | | | | | | | | | | | | | |
| 414 | History of chronic illness (confirmed by physician) | 1. Diabetes Mellitus 2. Hypertension 3. Kidney disease 4. Cardiac illness 5. Asthma 6. Not at all   Others specify -------- | | | | | | | | | | | | | | | |
|  | **Mothers knowledge towards ANC** |  | | | | | | | | | | | | | | | |
| 501 | Do you know women need to start ANC services before 3 months? | 1. Yes 2. No | | | | | | | | | | | | | | | |
| 502 | Do the pregnant with high blood pressure affect the fetus growth? | 1. Yes 2. No | | | | | | | | | | | | | | | |
| 503 | Does counseling on danger signs during pregnancy helps you to seek timely antenatal care? | 1. Yes 2. 2. No | | | | | | | | | | | | | | | |
| 504 | Does the pregnant woman need to test for HIV? | 1. Yes 2. No | | | | | | | | | | | | | | | |
| 505 | Does the pregnant women need to blood screen for Hgb? | 1. Yes 2.No | | | | | | | | | | | | | | | |
| 506 | Does the pregnant woman need to vaccinate for TT | 1. Yes 2.No | | | | | | | | | | | | | | | |
| 507 | Is you think pregnant women need iron tablet? | 1. Yes 2. No | | | | | | | | | | | | | | | |
| 508 | Does the pregnant woman need to be take ant parasitic drugs during pregnancy? | 1. yes  2. No | | | | | | | | | | | | | | | |
| 509 | Antenatal follow up helps pregnant women to identify complications in pregnancy? | 1. yes  2. No | | | | | | | | | | | | | | | |
| 510 | Health facility delivery is safer and better than home delivery? | 1. Yes 2. No | | | | | | | | | | | | | | | |
| 511 | The recommended place for delivery is at health facility? | 1. Yes 2. No | | | | | | | | | | | | | | | |
| 512 | It is recommended to have at least eight contacts during pregnancy? | 1. yes  2. No | | | | | | | | | | | | | | | |
| 513 | Antenatal care is recommended regardless of complications? | 1. Yes 2. No | | | | | | | | | | | | | | | |
| 514 | Delivery items should be prepared during the last weeks of pregnancy before delivery? | 1. yes  2. No | | | | | | | | | | | | | | | |
| 515 | Is family planning during pregnancy helps you to decide appropriate method after childbirth? | 1. yes  2. No | | | | | | | | | | | | | | | |
| 516 | Use of insect treated net prevents malaria acquisition? | 1. Yes 2. No | | | | | | | | | | | | | | | |
|  | **Attitudes of the mother on antenatal care** |  | | | | | | | | | | | | | | | |
|  | Items | 5.Strongly agree | | | | 4.Agree | | | 3.Neutral | | 2.Disagree | | | | 1.Strongly disagree | | |
| 601 | Would you agree, attained Antenatal follow-up is good to monitor your and your fetal health |  | | | |  | | |  | |  | | | |  | | |
| 602 | would your previous experience helps you to start antenatal care booking before 3 months if you became pregnancy |  | | | |  | | |  | |  | | | |  | |  |
| 603 | When you become pregnant would you go to ANC clinic for attending Antenatal care check-up important for me and my baby |  | | | |  | | |  | |  | | | |  | |  |
| 604 | In your previous ANC experience, would you allow to check vaginal examination for the next if it’s necessary |  | | | |  | | |  | |  | | | |  | | |
| 605 | In your previous ANC experience, would you modify your dietary habit as advice of professionals for your next pregnancy |  | | | |  | | |  | |  | | | |  | | |
| 606 | In your previous ANC experience would you take iron and folic acid Supplementations as prescribed of professional that’s important for you and your baby |  | | |  | | | |  | | | |  | | |  | |
|  | **Items for client satisfaction towards ANC** | | | | | | | | | | | | | | | | |
| 701 | The clinic site is easy to get | | | 1. Yes 2. No | | | | | | | | | | | | | |
| 702 | Waiting time was not too long | | | 1. Yes 2. No | | | | | | | | | | | | | |
| 703 | Clinic hours are convenient | | | 1. Yes 2. No | | | | | | | | | | | | | |
| 704 | Clinic area is clean | | | 1. Yes 2. No | | | | | | | | | | | | | |
| 705 | Information given about the method was sufficient | | | 1. Yes 2. No | | | | | | | | | | | | | |
| 706 | Privacy was maintained | | | 1. yes 2. No | | | | | | | | | | | | | |
| 707 | I have had sufficient consultation time to discuss about my needs | | | 1. yes 2. No | | | | | | | | | | | | | |
| 708 | Staff here are really friendly and respectful | | | 1. yes 2. No | | | | | | | | | | | | | |
| 709 | I would come back here if I need help again | | | 1. yes 2. No | | | | | | | | | | | | | |
| 710 | I would recommend the service to friends and relatives | | | 1. Yes 2. No | | | | | | | | | | | | | |
|  | **Women’s Decision making power** | | | | | | | | | | | | | | | | |
|  | Items | | 3.Respondent herself | | | | | 2.Respondent and husband jointly | | | | | | | 1.Husband only | | |
| 091 | Who usually decides how to spend the income that you bring into the household? | |  | | | | |  | | | | | | |  | | |
| 092 | Who usually decides how to spend the income that your partner brings into the household? | |  | | | | |  | | | | | | |  | | |
| 093 | Who usually decides about making smaller purchases, such as food and other less expensive needs? | |  | | | | |  | | | | | | |  | | |
| 094 | Who usually decides about making more expensive purchases, such as household equipment? | |  | | | | |  | | | | | | |  | | |
| 095 | Who usually decides on which family members you will visit and when? | |  | | | | |  | | | | | | |  | | |
| 095 | Who decides how many children you will have? | |  | | | | |  | | | | | | |  | | |
| 096 | Who usually decides whether your child will be taken for health care to a health facility when s/he is sick? | |  | | | | |  | | | | | | |  | | |
| 097 | Who usually decides whether you or your partner will use any types of contraception, such as condoms or pills? | |  | | | | |  | | | | | | |  | | |
|  | **Partner support** | |  | | | | | | | | | | |  | | | |
|  | Items | | 5.Strongly agree | | | | 4.Agree | | | 3.No opinion | | 2.Disagree | | 1.Stongly disagree | | | |
| 051 | I feel good about the amount of involvement my spouse has with my children. | |  | | | |  | | |  | |  | |  | | | |
| 052 | I am happy about the amount of interest that my spouse has shown in the children | |  | | | |  | | |  | |  | |  | | | |
| 053 | I am pleased with the amount of responsibility my spouse has taken for raising the children | |  | | | |  | | |  | |  | |  | | | |
| 054 | I am satisfied with my spouse's childbearing skills | |  | | | |  | | |  | |  | |  | | | |
| 055 | I am satisfied with the amount of time that my spouse can give to my children. | |  | | | |  | | |  | |  | |  | | | |
| 056 | My spouse usually does not help enough with the children | |  | | | |  | | |  | |  | |  | | | |
| 057 | My spouse thinks parenthood is an important and valuable part of life which pleases me greatly | |  | | | |  | | |  | |  | |  | | | |
| 058 | My spouse has sufficient knowledge about child development which seems to make him/her feel comfortable as a parent. | |  | | | |  | | |  | |  | |  | | | |
