# Supplementary material for: Level of optimal antenatal care utilization and its associated factors among pregnant women in Arba Minch town, southern Ethiopia: new WHO-recommended ANC 8+ model
Source: Front Glob Womens Health. 2024 Jul 16;5:1259637. doi: 10.3389/fgwh.2024.1259637 (PMC11286477; doi:10.3389/fgwh.2024.1259637)
Supplement: Supplementary file 2 [file Table2.docx]

## Annex I: - Informed Consent (ethical reference no: DD 1328/2022)

**Arba Minch University, College of Medicine and Health Science Department of Midwifery**

Name of institution____________________

Greeting. Hello, I am__________________. I am master’s student at Arba Minch University in the, College of Medicine and Health science. I would like to ask you a few questions about adequate antenatal care utilization. This will help us to improve the impact of lack of full antenatal care on our mothers, and their baby. Your name will not be written in this form and will never be used in connection with any information you tell us. All information given by you will be kept strictly confidential. Your participation is voluntary and you are not obliged to answer any question you do not wish to answer. If you feel discomfort with the interview please feel free to drop it any time you want. This interview will take about 20 minutes. Do I have your permission to continue? 1. If yes, continue to the next page 2. If no, skip to the next participant by writing reasons for his/ her refusal

Informed consent Certified by

Interviewer: Code_____________ Name__________________________ signature________

Date of interview____________ Time started___________ Time completed________

Result of interview: 1.Completed 2.Respondent not available 3.Refused 4. Partially completed

Checked by Supervisor: Name___________________ signature___________ Date_______

For any inconvenience and problem you can contact the principal investigator.

Dagne Deresa

Phone -0912143347 E-mails- [dagnederesa8@gmail.com](mailto:dagnederesa8@gmail.com)
